# Toxoplasmic encephalitis unmasked during treatment for miliary tuberculosis in a patient with human immunodeficiency virus infection: a case report and literature review

**DOI:** 10.1186/s41182-025-00852-z

**Published:** 2025-12-01

**Authors:** Yuta Kubono, Akira Kawashima, Takato Nakamoto, Fumiya Kawahara, Ryo Kuwata, Eri Inoue, Naokatsu Ando, Haruka Uemura, Daisuke Mizushima, Takahiro Aoki, Kisaburo Nagamune, Katsuji Teruya, Hiroyuki Gatanaga

**Affiliations:** 1 AIDS Clinical Center, National Center for Global Health and Medicine, Japan Institute for Health Security, 1-21-1 Toyama, Shinjuku ku, Tokyo, 162-8655 Japan; 2 Disease Control and Prevention Center, National Center for Global Health and Medicine, Japan Institute for Health Security, 1-21-1 Toyama, Shinjuku ku, Tokyo, 162-8655 Japan; 3 Department of Parasitology, National Institute of Infectious Diseases, Japan Institute for Health Security, 1-23-1 Toyama, Shinjuku ku, Tokyo, 162-8640 Japan; 4 Joint Research Center for Human Retrovirus Infection, Kumamoto University, 2-2-1 Honjo, Chuo-ku, Kumamoto City, Kumamoto 860-0811 Japan

**Keywords:** Human immunodeficiency virus, Opportunistic infection, *Toxoplasma gondii*, Toxoplasmic encephalitis, Polymerase chain reaction, Case report

## Abstract

**Background:**

Toxoplasmic encephalitis (TE) and tuberculoma are the leading causes of ring-enhancing brain lesions in patients with human immunodeficiency virus (HIV) infection. Because imaging findings of the two conditions overlap and cerebrospinal fluid (CSF) tests lack sensitivity, timely diagnosis is critical. Herein, we report the rare case of a patient with HIV infection and miliary tuberculosis who developed intracranial mass lesions after antiretroviral therapy (ART) initiation with high-dose prednisolone administration.

**Case presentation:**

A 53-year-old Nepalese man who had been living in Japan was diagnosed with HIV infection (CD4 count, 146 cells/µL) and miliary tuberculosis. Four-drug rifabutin-based therapy was initiated. However, trimethoprim–sulfamethoxazole (TMP–SMX) prophylaxis was discontinued because of a rash and replaced with monthly pentamidine. ART (dolutegravir/lamivudine) was initiated 3 weeks later. Prednisolone (60 mg/day) was administered for refractory tuberculous ascites, and the dose was tapered over 6 weeks. Eight weeks after ART, the patient developed a headache, and laboratory tests revealed a CD4 count of 384 cells/µL. Magnetic resonance imaging (MRI) revealed right frontal ring-enhancing lesions. CSF was acellular; polymerase chain reaction yielded negative results for several pathogens including *Mycobacterium tuberculosis* and positive results for *Toxoplasma gondii*. After a 5-day graded TMP–SMX desensitization, the patient received full-dose therapy for 6 weeks, followed by secondary prophylaxis. The patient’s headache resolved, and repeat MRI after 2 weeks revealed marked regression of the lesions. No radiological relapse was observed 3 months after treatment completion.

**Conclusions:**

TE can emerge during immune recovery at CD4 counts > 100 cells/µL when corticosteroid administration coincides with early ART. In patients receiving tuberculosis treatment who develop new brain lesions soon after ART, *T. gondii* polymerase chain reaction and prompt antiparasitic therapy should be pursued. Rifabutin permits concomitant use of dolutegravir, and TMP–SMX desensitization allows effective treatment and prophylaxis.

## Background

Toxoplasmic encephalitis (TE) is the leading central nervous system (CNS) opportunistic infection in patients with advanced human immunodeficiency virus (HIV) infection who do not receive appropriate prophylaxis [1]. The incidence of TE has decreased sharply in the antiretroviral therapy (ART) era because daily trimethoprim–sulfamethoxazole (TMP–SMX) administration for *Pneumocystis* pneumonia prophylaxis also prevents toxoplasmosis [2, 3]. TE is caused by the reactivation of latent *Toxoplasma gondii* cysts in the brain and usually develops when the CD4 + T-cell count decreases to < 100/µL [1]. The clinical manifestations of TE are usually subacute, with fever, headache, and neurological focal signs, such as confusion, seizures, and hemiparesis. Magnetic resonance imaging (MRI) typically reveals multiple ring-enhancing lesions with surrounding edema, most often in the basal ganglia or along the corticomedullary junction [4].

Tuberculoma of the central nervous system is a mass-forming granulomatous lesion resulting from hematogenous dissemination of *Mycobacterium tuberculosis* [5]. Tuberculoma can mimic TE radiologically [6] and symptomatically [7, 8]. Both entities remain common in regions where latent *T. gondii* and *M. tuberculosis* infections are endemic, and distinguishing them is clinically crucial because treatment differs [4, 9].

The introduction of ART can trigger immune reconstitution inflammatory syndrome (IRIS), which may unmask latent infections or worsen known ones, including TE or CNS tuberculosis (TB) [10]. In such cases, making an accurate diagnosis is challenging because the imaging features overlap, cerebrospinal fluid (CSF) studies lack sensitivity, and brain biopsy is rarely feasible.

Herein, we report the rare case of a patient with HIV infection and miliary TB who developed intracranial mass lesions after ART initiation and high-dose prednisolone administration.

## Case presentation

A 53-year-old Nepalese man, who had been living in Japan for more than a decade, presented with a 3-month history of progressive weight loss and intermittent fever. He reported no history of opportunistic infections or TB; and hypertension was the only comorbidity. Initial laboratory tests revealed HIV-1 infection, with a CD4 + T-cell count of 146 cells/μL and an HIV RNA viral load of 1 720 000 copies/mL. Contrast-enhanced computed tomography revealed diffuse micronodular infiltrates, necrotic cervical and axillary nodes, and large-volume ascites. Extensive microbiological evaluation including Ziehl–Neelsen stained smears, culture in Mycobacteria Growth Indicator Tubes, and nucleic acid amplification testing using the Xpert® MTB/RIF (Cepheid, Sunnyvale, CA, USA) platform revealed *M. tuberculosis* in multiple specimens, including axillary and cervical lymph-node aspirates, sputum, stool, and ascitic fluid. In addition, polymerase chain reaction (PCR)-based rifampicin resistance gene testing using the GeneXpert® MTB/RIF assay was negative. The patient was diagnosed with miliary TB with extrapulmonary involvement, specifically, tuberculous lymphadenitis, pulmonary TB, and tuberculous peritonitis.

Anti-TB therapy was initiated using isoniazid, rifabutin (chosen to reduce interactions with future ART), ethambutol, and pyrazinamide. At HIV diagnosis, the CD4 count (146 cells/µL) was > 100 cells/µL; however, the patient was seropositive for *T. gondii* IgG antibodies (122.5 IU/mL; cutoff, < 1.6 IU/mL); therefore, TMP–SMX (80 mg TMP/400 mg SMX) once daily was selected as the primary prophylaxis for *Pneumocystis jirovecii*. However, a generalized maculopapular rash on day 20 necessitated discontinuation of TMP–SMX and substitution with monthly pentamidine; atovaquone was avoided in view of rifabutin-mediated drug interactions.

Three weeks after starting anti-TB therapy, ART was initiated using dolutegravir and lamivudine coformulation; the CD4 + T-cell count on the day before ART initiation was 139 cells/µL, and the HIV RNA load was 462 000 copies/mL. Four weeks later, the CD4 count increased to 287 cells/μL. Drug susceptibility testing for *M. tuberculosis* was performed using the Bit Spectra SR system (Kyokuto Pharmaceutical Industrial Co., Ltd., Tokyo, Japan). Pyrazinamide susceptibility was determined using liquid culture media. The isolate was susceptible to isoniazid (0.2 µg/mL), rifampicin (40 µg/mL), ethambutol (2.5 µg/mL), and pyrazinamide. Based on these results, all four anti-TB drugs (isoniazid, rifabutin, ethambutol, and pyrazinamide) were continued without adverse effects. Prednisolone (60 mg/day; 1 mg/kg/day) was introduced 8 weeks into TB therapy for refractory tuberculous ascites that required repeated paracentesis; the dose was tapered by 10 mg weekly and discontinued after 6 weeks. After 74 days, the TB regimen was simplified to isoniazid and rifabutin, and the patient was discharged on day 82.

**Fig. 1:**
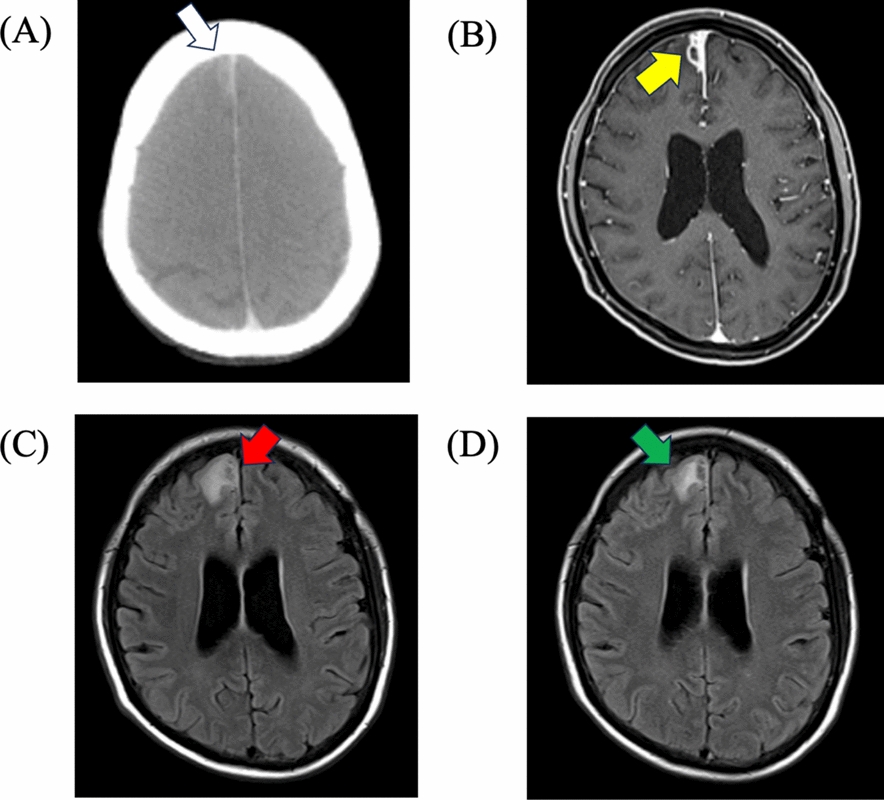
Axial imaging findings. **A** Brain computed tomography image shows a hyperdense lesion adjacent to the right falx cerebri (white arrow). **B** Contrast-enhanced T1-weighted magnetic resonance image shows cortical and parenchymal lesions in the right frontal lobe (yellow allow). **C** T2-weighed magnetic resonance image shows hyperintense edema surrounding the right side of the falx cerebri (red arrow). **D** Follow-up T2-weighted magnetic resonance image on day 14 showing reduced perilesional edema (green arrow)

Approximately 2 weeks after the first discharge, the patient developed persistent headaches and presented to the outpatient clinic. Follow-up contrast computed tomography performed to evaluate intra-abdominal TB lesions revealed a hyperdense lesion to the right of the falx cerebri (Fig. 1A). Subsequent contrast-enhanced brain MRI revealed ring-enhancing lesions with surrounding edema on the right side of the falx cerebri and on the cortical surface and parenchyma of the right frontal lobe (Fig. 1B, C).

On readmission, the patient was fully conscious and alert. His vital signs were as follows: temperature, 37.3 °C; blood pressure, 112/66 mmHg; heart rate, 93 beats/min; respiratory rate, 16 breaths/min; and oxygen saturation, 98% on ambient air. Neurological examination revealed no focal neurological deficits. No signs of meningeal irritation, including nuchal rigidity, Kernig’s sign, or Brudzinski’s sign, were observed. Laboratory testing revealed a C-reactive protein level of 1.58 mg/dL, CD4 + T-cell count of 384 cells/μL, and HIV RNA load of 24.6 copies/mL. CSF opening pressure (20 cmH2O) was normal, white blood cell count was 0 cells/µL, and the protein level was mildly elevated. CSF PCR testing for *M. tuberculosis*, Epstein–Barr virus, and John Cunningham virus and FilmArray® Meningitis/Encephalitis multiplex PCR panel (bioMérieux Japan Ltd., Tokyo, Japan) yielded negative results. Additionally, cytological examination and culture of the CSF revealed no bacteria including acid-fast bacilli. Real-time PCR targeting *T. gondii* 529-bp repeat element was performed based on a previous report [11]. The assay used a forward primer (Rep299F) with the sequence 5′-gtt ggg aag cga gag tc-3′ and a reverse primer (Rep432R) with the sequence 5′-att ctc tcc gcc atc acc ac-3′. Amplification was performed in a 20-µL reaction volume using PowerTrack™ SYBR Green Master Mix for qPCR (Thermo Fisher Scientific, MA) according to the manufacturer’s instructions. The reaction was performed using the StepOnePlus™ Real-time PCR system (Thermo Fisher Scientific, MA). The cycling conditions were as follows: initial denaturation at 95 °C for 20 s, followed by 40 cycles of denaturation at 95 °C for 3 s and annealing/extension at 60 °C for 30 s, followed by dissociation curve analysis at 60 °C to 95 °C. PCR result was positive in duplicate (Ct values, 36.53 and 37.02; positive control Ct value, 25.22). Although these values indicated a low parasite load, the amplification curves were well formed with clear melting peaks, suggesting a true-positive result.

High-dose dexamethasone, which was empirically administered for presumed tuberculoma, was discontinued. Graded TMP–SMX desensitization (from 0.4/2 mg to 80/400 mg) was performed for 5 days [12], and the patient was successfully transitioned to full-dose therapy (640 mg TMP/3200 mg SMX) daily without recurrence of rash. The headache improved within a week, and MRI on day 14 of hospitalization showed marked reduction in edema and lesion size (Fig. 1D). After completing 6 weeks of anti-toxoplasma therapy, the patient received secondary prophylaxis with TMP–SMX (320 mg TMP/1600 mg SMX) daily. At the 3-month follow-up, the patient remained asymptomatic, with a CD4 count of 522 cells/µL, undetectable HIV-1 RNA, and no radiological relapse.

## Discussion and conclusions

TE most commonly arises when CD4 counts fall below 100 cells/µL; however, our patient presented with radiologically and molecularly confirmed TE after immune recovery to 384 cells/µL. In this case, a rapid increase in the CD4 count was observed, and despite adequate viral suppression, the patient developed toxoplasmic encephalitis, which is considered quite unusual. The use of prednisone during the course of treatment influenced cellular immunity, contributing to the development of TE.

When CD4 counts fall below 100 cells/μL and the patient is seropositive for *T. gondii*, TMP–SMX (160 mg TMP/800 mg SMX) once daily is recommended [13]. In our patient, prophylaxis was initiated with 80 mg TMP/400 mg SMX once daily; however, TMP–SMX was discontinued early owing to adverse drug reactions. The use of high-dose prednisolone is a well-recognized independent risk factor for opportunistic infections in people with HIV infection [14]. In our patient, the administration of high-dose prednisolone to control refractory tuberculous ascites produced a transient, additional functional suppression of cell-mediated immunity; therefore, it almost certainly contributed to *T. gondii* reactivation despite a rising CD4 count. Since the patient exhibited no neurological symptoms at the time of HIV diagnosis, neuroimaging was not performed initially. However, this case suggests that in selected high-risk individuals—particularly those with positive serological test results for opportunistic pathogens or evidence of disseminated infections—pre-ART imaging should be considered, even in the absence of CNS symptoms.

The rapid increase in the CD4 count after ART created “unmasking” IRIS, in which a previously silent infection becomes clinically overt as antigen-specific responses return. Although cases of IRIS associated with TE are fewer than those associated with TB or cryptococcal meningitis [15], the short interval (12 weeks) between ART initiation and symptom onset, together with the prompt radiological response to antiparasitic therapy, supports this interpretation more convincingly than primary treatment failure for TB.

When the patient re-presented with new-onset headache, the differential diagnosis for ring-enhancing brain lesions included TE, tuberculoma or CNS lymphoma, fungal infections, and bacterial abscesses [4, 16]. TE is the most common cause of focal brain lesions in this population, typically presenting as multiple ring-enhancing lesions with surrounding vasogenic edema, often in the basal ganglia and gray–white matter junction [17]. Although tuberculomas may present with similar nodular lesions, typically distributed near the gray–white matter junction or adjacent to the ventricles [17], they are particularly common in patients with disseminated TB or from TB-endemic regions [18]. The diagnosis of TE in patients with HIV infection is generally presumptive, based on compatible imaging findings, clinical symptoms, positive *T. gondii* serology, and CD4 count < 100/μL [4]. Brain biopsy is the definitive diagnostic method but is often impractical because of procedural risks. In recent years, PCR testing of CSF has gained utility. Although its sensitivity is limited (ranging from 50 to 98%), its specificity is 100% [19]. Our patient was originally from Nepal, a country with a high TB burden, and had confirmed miliary TB, making tuberculoma a plausible diagnosis. However, the absence of systemic TB reactivation and rising CD4 counts warranted consideration of IRIS and alternative etiologies. Positive PCR results, serological evidence, and clinical response to treatment ultimately supported the diagnosis of TE.

The standard treatment for TE includes pyrimethamine, sulfadiazine, and leucovorin [20]. However, TMP–SMX is increasingly being recognized as an effective and accessible alternative [21]. Because our patient developed an allergic reaction to TMP–SMX during *Pneumocystis* pneumonia prophylaxis, pentamidine was selected as an alternative; however, it does not provide protection against TE. After TE diagnosis, desensitization was successfully performed [12], allowing full-dose TMP–SMX therapy and maintenance prophylaxis. Alternative regimens, such as atovaquone or clindamycin-based combinations, are viable options but have limitations, especially in patients on rifabutin [22]. Rifamycin drugs (especially rifampin; less so, rifabutin) increase UGT1A1 and P-gp activity, which lowers atovaquone levels by ≥ 34% [23]. From a tuberculosis perspective, rifabutin is preferred over rifampin to reduce interactions with dolutegravir, an HIV integrase inhibitor. Rifampin strongly induces CYP3A4 and can weaken antiretroviral therapy [24]. Current guidelines recommend rifabutin-based TB therapy in ART-naïve patients initiating dolutegravir-based regimens and underscore the importance of coordinating TB and HIV treatments [3]. This strategy preserved HIV suppression, achieved culture-negative miliary TB, and avoided interaction with anti-toxoplasma treatment and ART.

Although simultaneous TE and tuberculoma are uncommon, four cases have illustrated that overlapping manifestations occur in individuals with HIV infection in the past two decades (Table 1). Reported cases include thoracic spinal cord toxoplasmosis in a woman with HIV/TB coinfection [25], concurrent cerebral toxoplasmosis and miliary TB in a man with normal CD4 counts [26], and a Malaysian patient whose TE and miliary TB initially mimicked CNS lymphoma [27]. Another report described hemichorea-hemiballismus caused by TE in the subthalamic nucleus and cerebral peduncle [28]. These cases and the present case highlight the need for repeated diagnostic reassessment whenever new CNS lesions emerge during treatment because empirical anti-TB therapy alone may mask, but not eradicate, other opportunistic pathogens. Clinicians should strongly suspect TE in patients with HIV infection receiving corticosteroid therapy, even when the CD4 count is above the conventional threshold.

**Table 1:** Published human immunodeficiency virus-associated central nervous system coinfection by *Toxoplasma gondii* and *Mycobacterium tuberculosis*

Reference number	Year	Country	Age (y)	Sex	Presentation, CD4 (cells/µL)	Diagnostic basis	Imaging site	Treatment	Outcome
This case	2025	Japan	53	Male	Headache, 384	CSF PCR positive + serology + therapeutic response	Multiple cerebral lesions	Recovery on TMP–SMX + TB Rx	Alive
[25]	2017	Peru	33	Female	Paraplegia, 11	Serology + therapeutic response	Thoracic intramedullary ring lesion	Recovery on TMP–SMX + TB Rx	Alive
[26]	2022	Mali	56	Female	Fever, 446	Serology + therapeutic response	Multiple cerebral lesions + miliary TB	Improved on cotrimoxazole + HRZE	Alive
[27]	2013	Malaysia	31	Male	Seizures, 36	Therapeutic response	Multiple brain abscesses + miliary TB	Lesions resolved on TMP–SMX	Alive
[28]	2021	Philippines	24	Male	Hemichorea, 92	Serology + therapeutic response	Subthalamic + peduncular lesions	Marked recovery on dual therapy	Alive

*Abbreviations*: CSF, cerebrospinal fluid; PCR, polymerase chain reaction; TB, tuberculosis; TMP–SMX, trimethoprim–sulfamethoxazole; HRZE, isoniazid, rifampin, pyrazinamide, and ethambutol

In conclusion, this case highlights the diagnostic and therapeutic complexity of overlapping CNS opportunistic infections. A systematic approach that integrates evolving risk factors, focused molecular diagnostics, and drug-interaction-aware therapy can yield favorable outcomes, even when TE occurs during immune recovery after ART and miliary TB therapy.

## Data Availability

Data sharing is not applicable to this article as no datasets were generated or analyzed during the study.

## Abbreviations

TE: Toxoplasmic encephalitis
CNS: Central nervous system
HIV: Human immunodeficiency virus
ART: Antiretroviral therapy
TMP–SMX: Trimethoprim–sulfamethoxazole
MRI: Magnetic resonance imaging
IRIS: Immune reconstitution inflammatory syndrome
CSF: Cerebrospinal fluid
PCR: Polymerase chain reaction
TB: Tuberculosis
